# Insulin Therapy for Pre-Hyperglycemic Beta-Cell Endoplasmic Reticulum Crowding

**DOI:** 10.1371/journal.pone.0054351

**Published:** 2013-02-07

**Authors:** Afaf Absood, Benjamin Gandomani, Anthony Zaki, Vlad Nasta, Andrew Michail, Peter M. W. Habib, Israel Hodish

**Affiliations:** 1 Division of Metabolism, Endocrinology & Diabetes, University of Michigan Medical Center, Ann Arbor, Michigan, United States of America; 2 Wayne State University, Detroit, Michigan, United States of America; Broad Institute of Harvard and MIT, United States of America

## Abstract

Insulin therapy improves β-cell function in early stages of diabetes by mechanisms that may exceed alleviation of glucotoxicity. In advance type 2 diabetes, hyperglycemia causes β-cell damage and ultimately β-cell loss. At such an advanced stage, therapeutic modalities are often inadequate. Growing evidence indicates that in early stages of type-2 diabetes and some types of monogenic diabetes linked with malfunctioning endoplasmic-reticulum (ER), the β-cell ER fails to process sufficient proinsulin once it becomes overloaded. These changes manifest with ER distention (ER-crowding) and deficiency of secretory granules. We hypothesize that insulin therapy may improves β-cell function by alleviating ER-crowding. To support this hypothesis, we investigated pre-diabetic β-cell changes in hProC(A7)Y-CpepGFP transgenic mice that develop prolonged pre-diabetes due to proinsulin dysmaturation and ER-crowding. We attenuated the β-cell ER proinsulin synthesis with a treat-to-target insulin therapy while avoiding hypoglycemia and weight gain. Alleviation of ER-crowding resulted in temporary improvement in proinsulin maturation, insulin secretion and glucose tolerance. Our observations suggest that alleviation of pre-diabetic ER-crowding using a treat-to-target insulin therapy may improve β-cell function and may prevent further metabolic deterioration.

## Introduction

Insulin therapy is a widespread therapeutic modality for advance type 2 diabetes when other medications have failed to maintain reasonable glycemic control. A growing body of evidence identifies insulin therapy not only as a tool to improve glycemia, but also to preserve and recover endogenous β-cell function when introduced in early stages of the disease when pathophysiological mechanisms are potentially still reversible [1,2,3,4,5]. In both type-2 and type-1 diabetes, long term preservation of β-cell function has been shown to improve long term glycemic control [6,7], improve the response to medications (including insulin) [7], reduce the frequency of treatment-related hypoglycemia [7], and diminish complications [4,6,7,8,9,10]. Simply put, patients with higher residual β-cell function, regardless of medications used, have superior outcomes. It remains to be determined if insulin therapy can preserve β-cell function in a mechanism unrelated to alleviation of glucotoxicity, in the pre-hyperglycemic or pre-diabetic stage.

Pre-diabetes is the most common risk factor for type-2 diabetes. Patients with pre-diabetes demonstrate impaired fasting glucose, glucose intolerance or both, which may or may not progress to overt diabetes. Contrary to full-blown type-2 diabetes that encompasses progressive insulin deficiency, patients with pre-diabetes exhibit mild insulin insufficiency (the cause for borderline hyperglycemia) [11,12]. This condition affects approximately one third of the adult US population [13] and progresses to frank type-2 diabetes in about half of the cases. Once full-blown hyperglycemia develops, it imposes further metabolic injury on pancreatic β-cells (also known as “glucotoxicity”), that causes further insulin deficiency, additional hyperglycemia and ultimately results in β-cell demise [14,15]. The existing literature does not define a distinctive plasma glucose level that qualifies for glucotoxicity, yet mainly random glucose values exceeding 300 mg/dl (16.7 mmol/l) have been shown to cause β-cell damage [16,17,18]. At such an advanced stage, patients’ glycemic control with medications (including insulin therapy) is challenging and often inadequate [19]. Growing evidence identifies subtle secretory system defects in pancreatic β-cells during early stages of type-2 diabetes [20,21] and several types of monogenic diabetes linked with malfunctioning β-cell endoplasmic-reticulum (ER) (e.g., neonatal diabetes caused by proinsulin mutations [22], Mature Onset Diabetes of the Young type-4 or MODY4 [23], Wolcott-Rallison syndrome [24], Wolfram syndrome [25]). In these conditions the ER becomes distended (hereby referred to as “ER-crowding”) and secretory granules become heterogeneously scarce. ER-crowding is not present in over-fed animals without genetic predisposition to diabetes [23], thus cannot be ascribed to normal β-cell compensation. Of interest, among the genetic loci tied with type-2 diabetes in humans, the majority relate to the function of the endocrine pancreas while some are linked to increased plasma proinsulin to insulin ratios (e.g., TCF7L2, CDKAL1), indicating that type-2 diabetes develops in individuals genetically predisposed to develop β-cell dysfunction [26,27]. Although the causes of β-cell ER-crowding have not been identified, mathematical modeling have postulated that an overload of nascent proinsulin molecules beyond a certain individual threshold can alter the function of the crowded ER, cause proinsulin aggregation and reduce proinsulin output [28,29]. This putative mechanism may explain the similarities in β-cell secretory changes in early type-2 diabetes and neonatal diabetes that results from proinsulin mutations [20,22].

We have previously reported that the hProC(A7)Y-CpepPGFP transgenic mouse line is a useful model to investigate pre-diabetic ER-crowding [30]. In this line, a mildly expressed additional pre-proinsulin gene is labeled with the green-fluorescent-protein (GFP) and contains the *Akita* mutation. The latter disrupts the normal folding of a small fraction of the proinsulin molecules, causing the majority of the mice to have a prolonged impaired glucose tolerance and normal random glucose levels. Although this genetic defect has not been reported as a cause for type-2 diabetes in humans, the known etiology of its impaired glucose tolerance is of methodological benefit. Additionally, in this line, pancreatic islets share similar morphological features with extensively used rodent models of type-2 diabetes (e.g., LepR^db/db^ [20], New-Zealand Obese Mouse [31]) and its prolonged pre-diabetic phase resembles the human disease [20,21]. In hProC(A7)Y-CpepGFP transgenic mice, we have previously shown that ER-crowding already exists in the pre-hyperglycemic or pre-diabetic stage [30]. Since the mice are not overtly hyperglycemic, ER-crowding cannot be ascribed to glucotoxicity. In each pancreatic islet all β-cells are affected by ER-crowding, however only a fraction fail to produce and store insulin. By increasing β-cell mass, most animals evade frank diabetes.

In this article we investigate whether early treat-to-target insulin therapy that reduce proinsulin synthesis, can ameliorate pre-hyperglycemic β-cell ER crowding and improve its function.

## Experimental Procedures

### Animals

The hProC(A7)Y-CpepGFP transgenic mouse model was chosen to investigate pre-diabetic ER-crowding [30]. In this strain, the cause of ER-crowding is known and ascribed to a slight expression of a pre-proinsulin transgene labeled with GFP and inoculated with the *Akita* mutation (C(96)Y) [32]. The transgene is driven by the weak although highly specific Mouse-Insulin-Promoter-I [33], thus is being expressed at low levels and leads to impairment in glucose tolerance while random glucose is normal, a phenotype consistent with pre-diabetes. Conversely, when the mutation is highly expressed in the endogenous pre-proinsuln-II gene in the original *Akita* strain, males develop neonatal diabetes at the age of 4–8 weeks (male rodents are more susceptible to diabetes than females [34]). The hProCpepGFP strain that carries the wild type GFP-labeled proinsulin is processed and secreted normally, and therefore is used as a control [35]. LepR^db/db^ males develop early diabetes in an almost acute course at 6–8 weeks of age [16], and their pre-hyperglycemic phase (mild insulin insufficiency causing glucose intolerance) lasts only a week or two [36]. Therefore, this line could not have been used to investigate the affect of pre-hyperglycemic treat-to-target insulin therapy. Nonetheless, pair-fed (food intake is limited and compared to controls) B6.BKS(D)-LepR^db/j^ #000697 mice (hereby referred to as LepR^db/db^) from Jackson Laboratories (Bar Harbor, Main, USA) were used to correlate β-cell secretory changes to the ones found in hProC(A7)Y-CpepGFP.

Animals were housed in a pathogen-free facility on a 12-h light/dark cycle and fed a standard rodent chow. All experiments were performed in accordance with the regulations of the University of Michigan Committee on Use and Care of Animals (permit number - 10465). Unless otherwise stated, all experiments were performed in at least 3, 3–6 month-old male non-diabetic mice. Intraperitoneal glucose tolerance tests were performed on fasting animals with 1.0 mg dextrose per gram body weight. Insulin tolerance tests were performed on fasting animals with 0.00075 unit of Insulin Lispro from Eli Lilly® (Indianapolis, Indiana, USA) per gram body weight. Mice were euthanized by Isofluorane inhalation.

### Immunohistochemistry

Pancreata were fixed in 4% formaldehyde and embedded in paraffin. Five µm sections were de-paraffinized, progressively rehydrated and incubated with a self-prepared guinea-pig anti-insulin [30]; rabbit anti-BiP #3177 from Cell Signaling (Danvers, Massachusetts, USA); rabbit anti-CHOP #SC-575 from Santa Cruz (Santa Cruz, California, USA); and mouse anti-proinsulin from ALPCO (Salem, New Hampshire, USA). Following an interval blocking sessions, samples were incubated with the corresponding secondary antibodies, including AlexaFluor-555-conjugated goat anti-rabbit #A21429 from Invitrogen (Carlsbad, California, USA); AlexaFluor-647-anti-guinea-pig #A21450 from Invitrogen; and, AlexaFluor-488-conjugated rabbit anti-GFP #A21311 from Invitrogen. All fluorescence images were captured with EL6000 light microscope from Leica (Buffalo Grove, Illinoi, USA), equipped with Retiga 2000 monochrome camera from Qimaging (Surrey, British Columbia, Canada).

### Real-Time PCR (RT-PCR)

Pancreatic islets were isolated as we previously reported [35], using Hanks’ balanced salt solution (HBSS) #14175 from GIBCO, Invitrogen; and Collagenase-P from Roche Diagnostics (Indianapolis, Illinoi, USA). Freshly isolated islets were homogenized in 1% SDS lysis buffer for protein extraction or processed with the RNeasy Plus kit from Qiagen (Valencia, California, USA) for RNA extraction. cDNA from the RNA template was generated using SuperScript III first-strand reverse transcriptase from Invitrogen with random hexamers. cDNA samples were then amplified in a real-time fluorescence thermal cycler using appropriate primers and thermal cycles as we previously reported [30]. mRNA was normalized to β-actin and transcript levels were calculated using the comparative threshold cycle (CT) method (2^−ΔCT^).

### Western Blotting

Islets were boiled in 1% SDS lysis buffer containing 100 mM dithiothreitol (DTT). Samples were normalized to dsDNA using the Qubit system from Invitrogen. Proteins were resolved by 4%–12% acrylamide gradient SDS-PAGE gels and electrotransferred to polyvinylidene (PVDF) membranes. After blocking, membranes were probed with unconjugated rabbit anti-GFP #A11122 from Molecular Probes, Invitrogen; mouse anti α-tubulin #T5168 from Sigma (St. Louis, Missouri, USA); mouse anti-insulin #I2018 from Sigma, and rabbit anti-BiP #3177 from Cell Signaling. WesternDot 625 kit form Invitrogen was used to detect labeled bands by incubation with the suitable biotinylated secondary antibodies. In this system, insulin and proinsulin bands were corroborated to be sensitive to subtle differences in peptide levels.

### Elisa Assays

Elisa assays for mouse insulin #80-INSMSH-Eol, mouse proinsulin #80-PINMS-Eol, and mouse C-peptide #80-CPTMS-Eol were from ALPCO.

### Transmission Electron Microscopy

Pancreatic tissues from pre-diabetic hProC(A7)Y-CpepGFP mice and wild-type controls were fixed in buffered glutaraldehyde, postfixed in OsO_4_, and dehydrated with graded alcohols and propylene oxide. After being embedded in Spurr’s resin, 70 nm sections were gridded and stained with uranyl acetate and lead citrate. Philips CM-100 electron microscope was used for scanning.

### Statistical Analysis

Normality was assessed by Shapiro-Wilk test. Differences between means were determined by ANOVA and two-tailed *Student* t-test or, if non-normally distributed by the Kruskal-Wallis and Wilcoxon test, respectively. Results are presented as mean ± standard error of the mean (SEM). P-value≤0.05 was defined as statistically significant and p-values ranging from 0.1 to 0.05 were defined as a trend toward significance. The Prism software package (Madison, Wisconsin, USA) was used for statistical analysis.

## Results

### Similar Proinsulin Maturation Defects are Found in Pre-diabetic hProC(A7)Y-CpepGFP and LepR^db/db^ Mice

**Figure 1:**
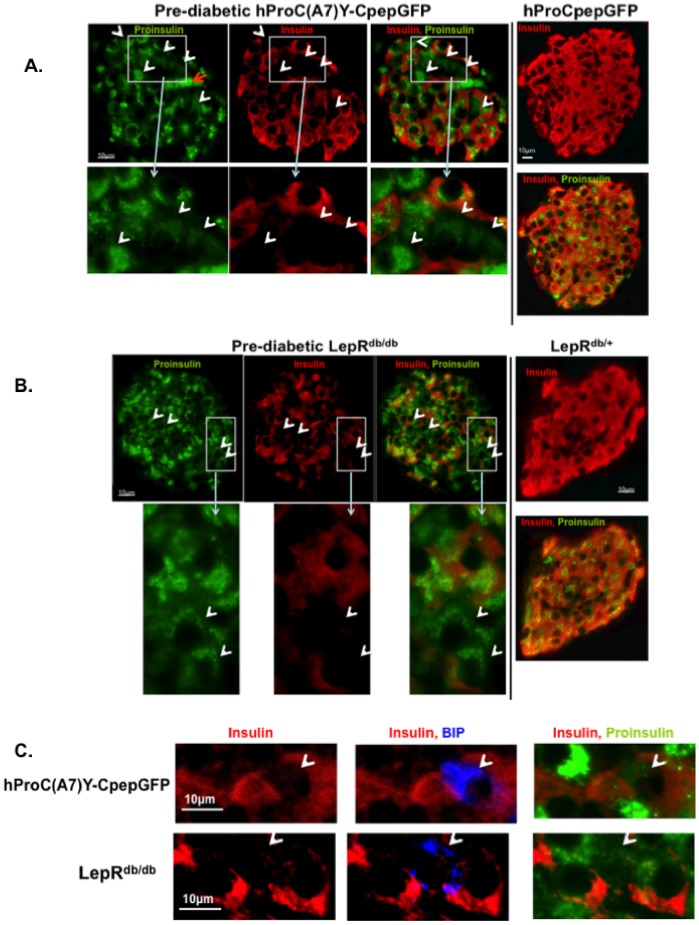
Distribution of insulin and proinsulin in pancreatic islets of pre-diabetic mice. Sections of paraffin embedded pancreata were immunostained with anti-insulin antibodies (red), anti-proinsulin antibodies (green) and anti-BIP (blue). The endogenous GFP fluorescence was quenched prior immunostaining. A. Endogenous insulin and proinsulin in pre-diabetic hProC(A7)Y-CpepGFP male compared to hProCpepGFP control. B. Endogenous insulin and proinsulin in pre-diabetic LepR^db/db^ male compare to LepR^db/+^ control. C. β-cell BIP in pre-diabetic hProC(A7)Y-CpepGFP and LepR^db/db^ males. In genetically predisposed pre-diabetic mice, maturation of proinsulin to insulin is heterogeneously compromised, likely in β-cells more affected by the ER insult (white arrowheads). Red arrow denotes autofluorescence deriving from red blood cells.

We first sought to determine if similar secretory system changes occur in pre-diabetic hProC(A7)Y-CpepGFP and LepR^db/db^ transgenic mice. The latter has been extensively used to model full blown type 2 diabetes but due to rapid development of hyperglycemia (contrary to hProC(A7)Y-CpepGFP transgenic mice) it is less suitable to investigate pre-diabetes. We have previously reported that in each pancreatic islet of pre-diabetic hProC(A7)Y-CpepGFP transgenic mouse, all β-cells are affected by ER-crowding, but the severity of this process is uneven [30]. In each islet about a third of β-cells exhibited a more advanced dysfunction and failed to store mature insulin, likely due to the chronic nature of the disease, inhomogeneous ER insult, and inhomogeneous β-cell age. These insulin deficient cells were the ones more affected by ER insult, evidenced by greater accumulation of misfolded GFP-labeled proinsulin. In Western-blotting experiments with isolated islets, we have demonstrated that the small amount of mutant GFP-labeled proinsulin (1∶1,000−1∶10,000 of the total) perturbed the production and maturation of the endogenous proinsulin [30,35]. Immunohystochemistry using specific antibodies against insulin and proinsulin showed that most insulin deficient β-cells in each hProC(A7)Y-CpepGFP islet synthetize proinsulin in ER and golgi patterns (Figure 1A, white arrowheads) regardless of the content of mature insulin. In control hPrCpepGFP islets that express wild-type GFP-labeled proinsulin, all β-cells exhibited both proinsulin and mature insulin (Figure 1A; right panel). Of interest, the antibody used against endogenous proinsulin did not react with GFP-labeled proinsulin (not shown). These results together with our previous findings [30] suggest that pre-diabetes in hProC(A7)Y-CpepGFP mice is associated with impairment of endogenous proinsulin maturation even than the insult occurs in the GFP-labeled proinsulin.

We explored the possibility that similar heterogeneous proinsulin dysmaturation occurs in islets of 8-week-old pair-fed LepR^db/db^ male mice that still uphold normal random glucose (for a short period) and thus expected to be in the pre-hyperglycemic stage. Immunohystochemistry showed that similar to pre-diabetic hProC(A7)Y-CpepGFP transgenic mice, a number of β-cells in each pre-diabetic LepR^db/db^ islet were deficient of mature insulin yet produced proinsulin in an ER and golgi patterns (Figure 1B, white erowheads). Similar to hProCpepGFP, LepRdb/+ controls exhibited homogenous distribution of insulin (Figure 1B: right panel). Both in pre-diabetic hProC(A7)Y-CpepGFP and LepR^db/db^ males, the ER chaperone Immunoglobulin Heavy Chain Binding Protein (BIP) was stained positive in insulin empty cells and was negative in β-cells stained positive for insulin (Figure 1C) or hProCpepGFP and LepR^db/+^ controls (not shown).

In summary, the data shows that similar β-cell phenotypic defects (heterogeneous impairment of proinsulin maturation) can be found in pre-diabetic hProC(A7)Y-CpepGFP and LepR^db/db^.

### Treat-to-Target Insulin Therapy Suppressed Proinsulin Synthesis in Pre-Diabetic Mice while Evading Hypoglycemia and Weight Gain

**Figure 2:**
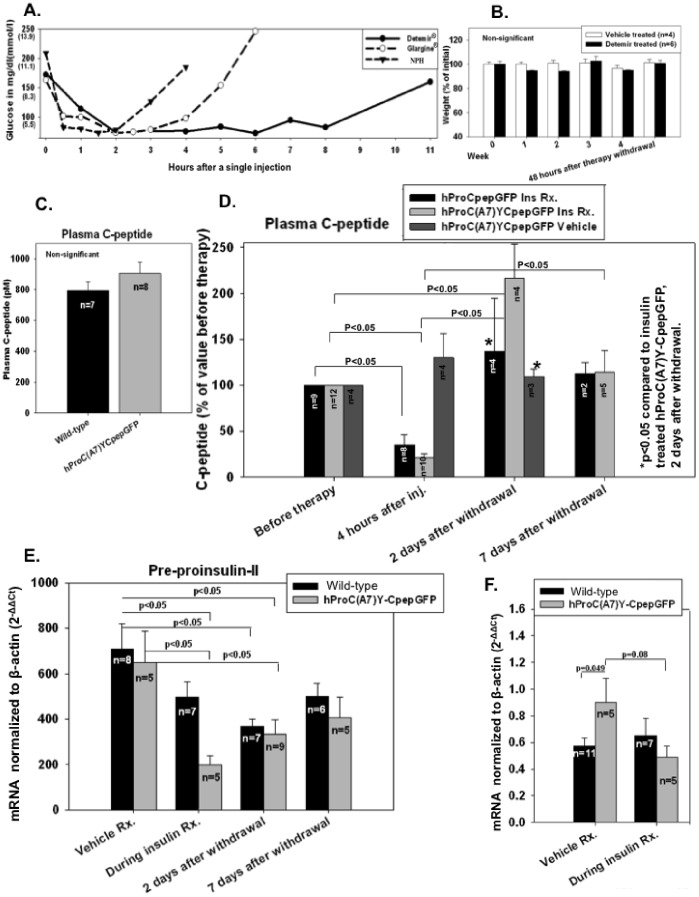
Effect of a treat-to-target insulin therapy on insulin production. Mice were treated with a treat-to-target insulin therapy for a month and tissues were harvested for mRNA and protein, before and after drug washout. A. Pharmacodynamic profiles of different insulin formulations following a single subcutaneous injection. B. Mean weight during therapy. C. Pre-treatment plasma C-peptide. D. Plasma C-peptide changes in insulin treated hProCpepGFP, insulin treated wild-type mice controls, and vehicle treated hProC(A7)Y-CpepGFP. E. Pre-proinsulin–II mRNA message normalized to β-actin, depicted as mean 2^−ΔCT^ ± SEM. F. BIP mRNA message. In pre-diabetic hProC(A7)Y-CpepGFP mice a treat-to-target insulin therapy suppressed proinsulin production, yet improved insulin secretion after therapy withdrawal.

We thought to investigate whether insulin therapy ameliorate pre-diabetic β-cell ER crowding and improve its function. Pre-diabetic hProC(A7)Y-CpepGFP transgenic mice were utilized because their genetic β-cell insult is known, the length of their pre-diabetes is as prolonged as in the human disease (contrary to LepR^db/db^), similar β-cell secretory changes are found in type 2 diabetes, and the confounding effect of glucotoxicity can be excluded. These mice are not obese, and insulin sensitive compared to wild-type controls (not shown). We have developed a treat-to-target insulin therapy, aiming to reduce β-cell proinsulin sybnthesis while avoiding hypoglycemia. This was achieved by giving twice daily subcutaneous injection of insulin Detemir® (Novo-Nordisk, Bagsvaerd, Denmark) that conferred the most suitable pharmacodynamic profile (Figure 2A). As mice graze almost continuously through the day [37], this regimen was expected to have satisfied both basal and prandial insulin requirements. The influence of Detemir was monitored with glucose readings taken 4 hours after injections during its peak pharmacodynamic effect (Figure 2A). Therapy goal was set at 95–110 mg/dl or 5.3–6.1 mmol/l (4 hours after injection) and insulin dosage was adjusted twice weekly to compensate for expected fluctuations in insulin requirements [38]. Insulin therapy was administered for a period of 4 weeks, to allow full regeneration of the endocrine pancreas [39]. Mice weight during the therapy was stable and comparable to vehicle treated controls (Figure 2B).

We then measured plasma C-peptide at different time points to document suppression of endogenous insulin secretion (C-peptide can only be derived from endogenous insulin). As depicted in Figure 2C, similar plasma C-peptide were detected in plasma samples of hProC(A7)Y-CpepGFP and wild-type controls. As shown in Figure 2D, Detemir injection suppressed plasma C-peptide (4 hours after injection) by approximately 70% (within the detectability range of the assay) both in pre-diabetic hProC(A7)Y-CpepGFP and wild-type controls. Forty-eight hours after the last Detemir injection, when the drug was expected to have fully metabolized [40], plasma C-peptide was at least as high as in the untreated mice (Figure 2D) while random glucose levels were similar to vehicle treated controls (not shown). Accordingly, 48 hours washout was chosen to assess endogenous proinsulin production and maturation when a full endocrine recovery was assumed.

### Suppression of Proinsulin Synthesis Alleviated ER-Crowding

We measured transcriptional message (mRNA) of pre-proinsulin during and after insulin therapy by RT-PCR with specific primers against pre-proinsulin-II (responsible for the majority of the proinsulin transcript in rodents [41]). As shown in Figure 2E, both pre-diabetic hProC(A7)Y-CpepGFP transgenic mice and wild-type controls showed that pre-proinsulin mRNA was repressed not only during therapy but also 48 hours after drug washout, despite complete functionality of the endocrine pancreas (Figure 2D). This was partially restored a week after withdrawal (Figure 2E). We studied the effect of ER-crowding alleviation on the ER stress response pathway by measuring the transcriptional message of the Immunoglobulin Heavy Chain Binding Protein (BIP). BIP is a chaperone protein in the lumen of the ER and a sensitive marker for ER stress [42]. We previously showed that mRNA message of BIP is elevated in pre-diabetic hProC(A7)Y-CpepGFP transgenic mice compared to Wild-type controls [30]. During insulin therapy, BIP mRNA in pre-diabetic hProC(A7)Y-CpepGFP was lower and comparable to wild-type controls (Figure 2F).

**Figure 3:**
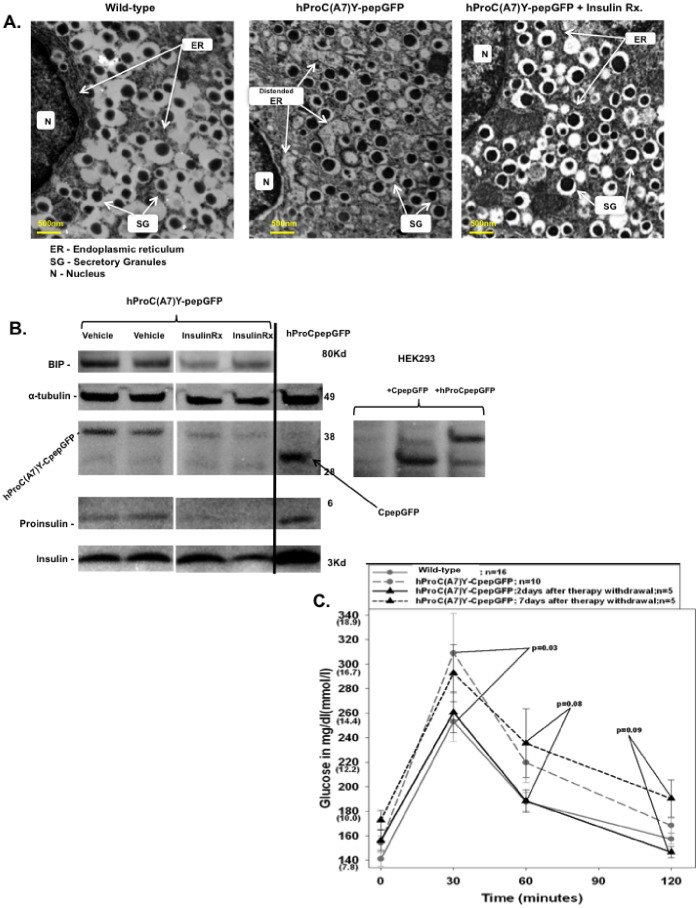
Effect of a treat-to-target insulin therapy on ER crowding, proinsulin maturation and glucose tolerance. Mice were treated with a treat-to-target insulin therapy for a month and tested with intraperitoneal glucose tolerance test. Pancreatic sections were imaged with a transmission electron microscope and pancreatic islets were collected 48 hours after therapy withdrawal. A. Electron microscope images of insulin producing β-cells in pre-diabetic hProC(A7)Y-CpepGFP mice (insulin Detemir and vehicle treated) compared to wild-type controls. B. Western blotting of islets lysate in reducing SDS-PAGE gel showing the steady state levels of endogenous proinsulin (proinsulin), endogenous insulin (insulin), GFP-labeled mutant proinsulin (hProC(A7)Y-CpepGFP), fully processed GFP labeled C-peptide (CpepGFP), BIP, and α-tubulin for internal control. The lower panel shows graphic representation of endogenous insulin/proinsulin ratio. Three samples were used from each group. Representative samples are from two different vehicle treated pre-diabetic hProC(A7)Y-CpepGFP mice (left panel) and two different insulin treated pre-diabetic hProC(A7)Y-CpepGFP mice (middle panel). hProCpepGFP transgenic mouse and transfected HEK293 cells were used as controls to identify the location of specific bands. C. Intraperitoneal glucose tolerance test 48 hours and a week after insulin withdrawal in the same mice. Alleviation of ER crowding increased endogenous insulin/proinsulin ratio in pre-diabetic hProC(A7)Y-CpepGFP mice and temporarily normalized glucose tolerance.

Transmission electron microscopy revealed normalization of ER diameter in insulin producing β-cells of Detemir treated pre-diabetic hProC(A7)Y-CpepGFP transgenic mice, compared to vehicle treated controls (Figure 3A). The immunohistochemical distribution of insulin, proinsulin, and BIP did not remarkably change during therapy (not shown). We then studied if attenuation in endogenous proinsulin synthesis in pre-diabetic hProC(A7)Y-CpepGFP transgenic mice can alleviate the ER from accumulating misfolded GFP-labeled proinsulin. Islets lysate was resolved in SDS gel to separate the endogenous proinsulin and insulin from GFP-labeled misfolded proinsulin. As depicted in Figure 3B, 48 hours after Detemir withdrawal misfolded GFP-labeled proinsulin was lower than vehicle treated controls and the steady state ratio between endogenous insulin and proinsulin tended to increase (Figure 3B), although not in a statistical fashion (11.6±2.9 versus 5.2±0.3). This data indicated that reduction in ER-crowding may have improved endogenous proinsulin maturation.

### Alleviation of ER-Crowding in Pre-diabetic hProC(A7)Y-CpepGFP Transgenic Mice Improved Insulin Secretion and Glucose Tolerance

To investigate the clinical impact of ER-crowding alleviation, we preformed intraperitoneal glucose tolerance test in pre-diabetic hProC(A7)Y-CpepGFP mice 48 hours and a week after Detemir washout. Plasma C-peptide improved (Figure 2D) and glucose tolerance normalized compared to vehicle treated controls (Figure 3C). In wild-type controls, plasma C-peptide did not significantly change after therapy withdrawal (Figure 2D). Although hProC(A7)Y-CpepGFP transgenic mice did not gain weight during the therapy (Figure 2B) and did not develop hypoglycemia after therapy withdrawal (not shown), plasma C-peptide level almost doubled 2 days after insulin therapy withdrawal (Figure 2D). This phenomenon may partially be explained by relatively high plasma proinsulin level in pre-diabetic hProC(A7)Y-CpepGFP mice compared to controls (12.68±2.3 pM in hProC(A7)Y-CpepGFP versus 4.0±0.7 in wild-type controls; p = 0.05). Proinsulin is about 20 times less biologically active than fully mature insulin [43].

Likely due to a relapse in ER-crowding a week after therapy withdrawal, insulin secretion (plasma C-peptide) decreased to the pre-treatment range and glucose tolerance deteriorated (Figures 2D and 3C). Akin the mRNA pattern (Figure 2F) BIP protein content was suppressed after insulin therapy (Figure 3B).

Collectively, our data shows that suppression of ER-crowding by attenuating proinsulin synthesis resulted in temporary improvement in insulin secretion.

## Discussion

Although obesity and chronic insulin resistance have been considered major risk factors for type-2 diabetes, only less than one fifth of obese individuals develop diabetes [44]. Likewise, insulin sensitivity is not necessarily higher in patients with diabetes, compared to non-diabetic obese [45], indicating that obesity and insulin resistance likely function as environmental stressors to genuinely defective β-cells in genetically predisposed individuals. In effect, about 20% of patients with type 2 diabetes in the western world are lean and relatively insulin sensitive [46], a condition that has become widespread in the developing world [47]. Thereby, β-cell dysfunction is considered a fundamental element in the development of type-2 diabetes. Of interest, the majority of the genetic polymorphism associated with type-2 diabetes is localized within genes encoded in the endocrine pancreas (reviewed in [48]), but their role in early pre-diabetic β-cell dysfunction has not been entirely elucidated. Mathematical modeling has indicated that crowding of the ER with nascent proinsulin molecules beyond an individual threshold may slow its ER transit time, perturb its maturation, cause proinsulin aggregation and diminish its output to the secretory granules unrelated to the primary cause of ER-crowding [28,29]. This mechanism may explain the association between pre-diabetes in humans, LepR^db/db^, and hProC(A7)Y-CpepGFP transgenic mice. It is not unlikely, that a broad array of pathogenetic mechanisms cause ER-crowding in humans, including anomalous ER chaperons, oxidoreductases, membrane transporters, or other ER residents. Consequently, the severity and the number of ER offenders in each individual predict the severity and the amount of environmental stressors needed to uncover pre-diabetes.

Insulin therapy has been extensively used for the management of advance type-2 diabetes in humans, when the endocrine pancreas is exceedingly deficient and patients are overtly hyperglycemic. In this stage, only a third of the patients treated with insulin achieve the therapy goal (A1C<7%) [19] and the remainder becomes susceptible to complications. Though insulin sensitizers (i.e., biguanides [49] and thiazolidinediones [50]) have been shown to slow the progression of pre-diabetes to some extent, these agents have an upper dosage limitation and thus cannot be up titrated when its biological effect weakens. Insulin is advantageous by the absence of upper dosage limitation. Indeed, new evidence from human studies indicated that basal insulin therapy can extend the pre-diabetic stage without significant increment in risk [51]. In this manuscript, we report the effect of a treat-to-target insulin therapy on β-cell function of pre-diabetic animals while excluding the confounding effect of glucotoxicity alleviation. Insulin therapy has been utilized to investigate its effect on β-cell function and ER-stress in rodent models. Yet, in these studies animals (or controls) were hyperglycemic, thus the beneficial effect of the therapy could not have been discerned from alleviation of β-cell glucotoxicity [52,53,54]. Also, in these models insulin was given as a relatively short-acting formulation or in fixed dosage rather than in a goal directed manner (i.e., treat-to-target insulin therapy as used in humans) that endorses adequate monitoring and dosage titrations.

Our results revealed that similar alterations can be found in a variety of pre-diabetic animals (hProC(A7)Y-CpepGFP and LepR^db/db^), namely heterogeneous impairment in proinsulin maturation (Figure 1) and ER distention [30], implying that ER-crowding may function as a common pathophysiological mechanism in the development of pre-diabetes irrespective to the pathogenetic cause of ER-crowding. ER-crowding is not present in over-fed animals without genetic predisposition to diabetes [23], thus cannot be ascribed to normal β-cell compensation. We attenuated proinsulin synthesis in β-cells of pre-diabetic hProC(A7)Y-CpepGFP transgenic mice with a treat-to-target insulin therapy. This manipulation was not expected to ameliorate insulin secretion unless alleviating a β-cell defect. This method facilitated meticulous suppression of endogenous proinsulin synthesis without exposing the animals to hypoglycemia or weight gain (Figures 2B and 2D). We investigated β-cell function and endogenous insulin secretion 48 hours after therapy withdrawal, when the ER was decongested from misfolded GFP-labeled proinsulin and endogenous proinsulin (Figures 3A and 3B) and animals were fully dependent on their own insulin secretion to maintain euglycemia (Figure 2D). Similar patterns of pre-proinsulin mRNA suppression were noted in pre-diabetic hProC(A7)Y-CpepGFP and wild-type controls (Figure 2E). In pre-diabetic hProC(A7)Y-CpepGFP transgenic mice the therapy alleviated the ER insult (Figure 3B) and crowding (Figure 3A) and thus showed improved insulin secretion (Figure 2D) and transiently normalized glucose tolerance (Figure 3C). Based on our prior studies [30,35], we propose that a decline in ER-crowding and proinsulin aggregation improved proinsulin maturation (increased insulin to proinsulin ratio), consequently improved insulin production and insulin secretion. This effect could only be documented before the ER was likely repopulated with the misfolded GFP-labeled proinsulin about a week after therapy withdrawal (Figures 2D and 3C). As we previously reported [30], BIP is up regulated in hProC(A7)Y-CpepGFP (Figure 2F), particularly in β-cells more affected by the insult (Figure 1C). This was alleviated with insulin therapy (Figures 2F and 3B), likely due to down regulation of the ER machinery. During therapy, BIP mRNA message was similar in both pre-diabetic hProC(A7)Y-CpepGFP mice and wild-type controls (Figure 2F).

Pending on further investigation in humans, we anticipate that alleviation of ER-crowding may enhance preservation of β-cell function and prevent further deterioration of the β-cells. We propose that currently emerging approaches [51] to provide treat-to-target insulin therapy during the pre-diabetic stage in order to suppress endogenous insulin production and ER-crowding, may benefit patients with pre-diabetes without significant rise in treatment related risk.
